# A study of hydrogen plasma-induced charging effect in EUV lithography systems

**DOI:** 10.1186/s11671-023-03799-4

**Published:** 2023-02-23

**Authors:** Yao-Hung Huang, Chrong Jung Lin, Ya-Chin King

**Affiliations:** Institute of Electronics Engineering, National Tsing Hua University, Hsinchu, Taiwan

**Keywords:** Extreme ultraviolet (EUV), Lithography, EUV-induced hydrogen plasma

## Abstract

In the extreme ultraviolet lithography system, EUV-induced hydrogen plasma charging effect is observed by in situ embedded micro-detector array. The 4k-pixel on-wafer array can detect and store the distributions of H_2_ plasma in each in-pixel floating gate for non-destructive off-line read. The local uniformity of H_2_ plasma intensity extracted by the threshold voltages on an array and its distributions across a wafer by the average bit cell current of MDAs provide insights into the detailed conditions inside advanced EUV lithography chambers.

## Introduction

Extreme ultraviolet (EUV) lithography [1–3] is an optical patterning technology adopted in semiconductor processes for 7 nm technology node and beyond [4]. A CO_2_ laser creates EUV radiation with a wavelength of 13.5 nm by shooting two consecutive laser pulses on a Sn droplet. The first low-intensity pulse flattens the Sn droplet into a pancake shape. The second high-intensity laser pulse vaporizes the Sn droplet to generate a plasma that emits EUV light [5]. During the de-excitation of the Sn plasma, some Sn atoms may accumulate on the incidence mirrors within the chamber, reducing their reflectivity. To maintain the mirrors’ cleanliness, hydrogen gas is introduced to react with the Sn atoms, forming SnH_4_ in a gaseous form, which can be easily removed through pumping [6, 7].

Hydrogen gas is transformed into high-density plasma under high-intensity EUV light, which ionizes the hydrogen gas, forming plasma that consists of ions, electrons, and neutral atoms. EUV light also excites the hydrogen atoms into higher energy levels. The interactions between the charges and neutral atoms determine the dynamics of the plasma, including its temperature, density, and spectra [8–13]. In the commercial EUV lithography scanner [14–17], the EUV-induced hydrogen plasma needs to be investigated to understand its effect on the reliability and yield of the Si chips [18, 19].

EUV-induced hydrogen plasma can cause undesirable effects on the devices/circuits during lithography processes. Plasma-induced damage (PID) due to high-energy ions bombardment and charging damage induced by conduction current from plasma has been studied for structures that experience plasma-related treatments during manufacturing [20, 21]. PID can lead to degradations of device parameters such as threshold voltage, dielectric leakage current, and transconductance. As a result, detecting the plasma distribution and the charging effect during the process is essential. In this study, the EUV-induced hydrogen plasma charging effect is first-time reported and monitored by in situ embedded micro-detector array (MDA) [22]. The micro-detector's threshold voltage (V_th_) and bit cell current (BCC) are used to analyze the magnitude and distribution of hydrogen plasma in the EUV lithography chambers.

## Micro-detection array and operation principle

**Fig. 1:**
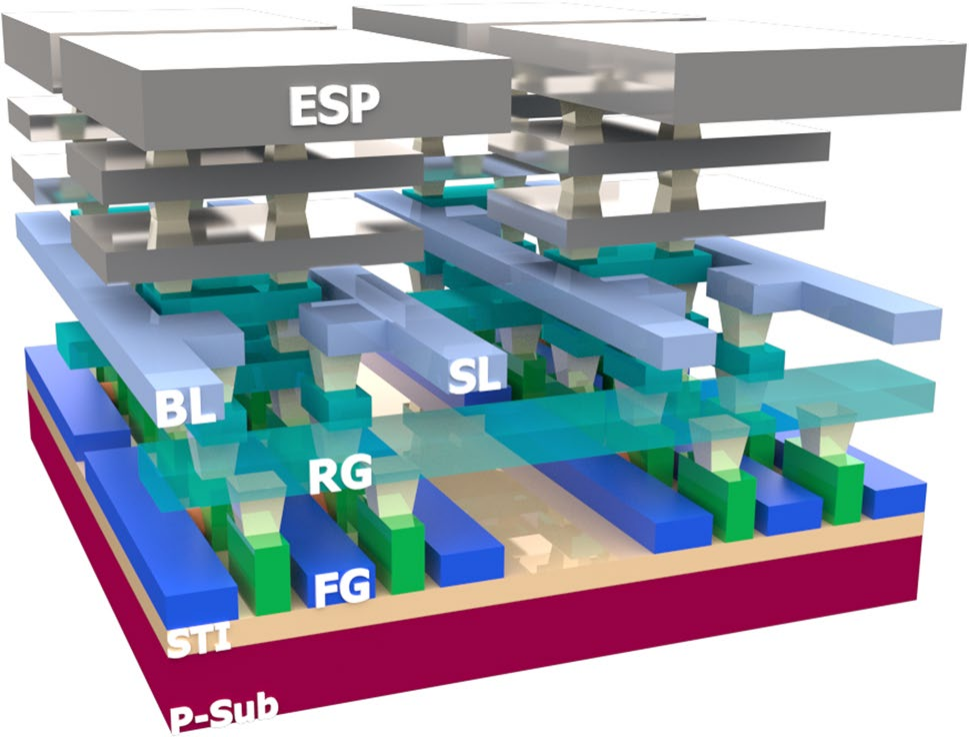
3D structure of a 2 × 2 MDA

A FinFET-compatible MDA can monitor the EUV-induced plasma in the EUV lithography system. Figure 1 is the 3D structure of a 2 × 2 MDA, and a 4k-pixel array is fabrication on-wafer for follow-up experiments. First, each micro-detector comprises an Energy Sensing Pad (ESP) extended to the wafer surface and an n-channel floating-gate (FG) transistor for data storage and readout. ESPs are connected to the surface Al pads in the standard logic process, so they can collect charge induced by H_2_ plasma in the EUV chamber. During the charging of ESPs during EUV processes, the potential of ESP is coupled to the FG by a laterally capacitively coupling structure between the contact slot and the FG. The coupled voltage from ESP will lead to changes in FG charge. To read out the stored FG charge, the source line (SL) and the bit line (BL) are connected to the source and drain terminal of the floating-gate-controlled n-channel FinFET, respectively. Through another coupling gate, read gate (RG), the channel current from SL to BL can then be measured through off-line wafer level tests.

**Fig. 2:**
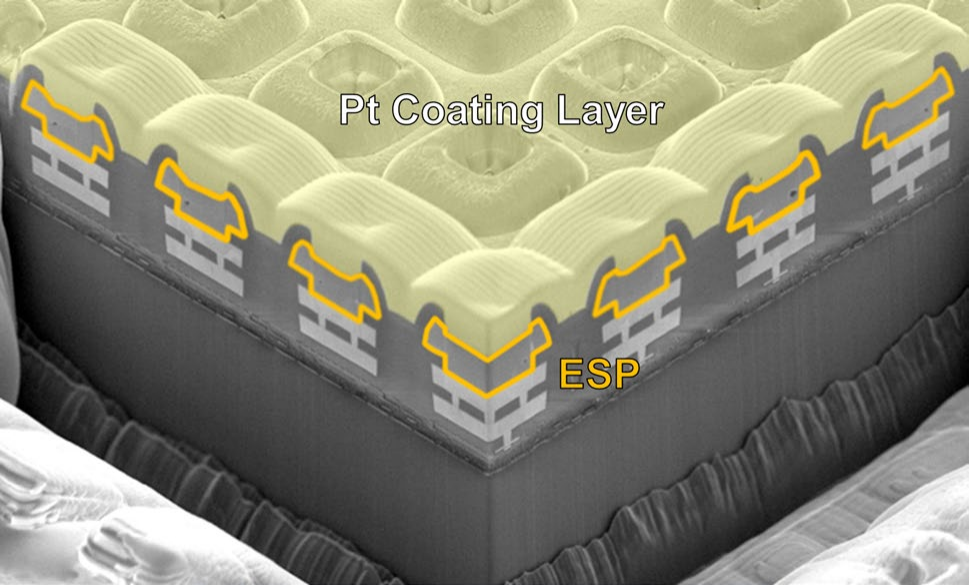
SEM cross section of 4k-pixel MDA where ESPs are placed on the wafer surface. The MDA is coated with Pt to increase the SEM signal-to-noise ratio

Figure 2 is the Scanning Electron Microscope (SEM) cross-sectional view of a 4k MDA sample, where the ESPs are highlighted in yellow. The ESPs are directly connected to the top metal of the back end of line (BEOL) structure. In the SEM image, a Pt layer is coated on the samples to increase the SEM image's signal-to-noise ratio [23], which is not presented for regular detection in EUV chambers.

## H_2_ plasma charging effect in EUV chamber

**Fig. 3:**
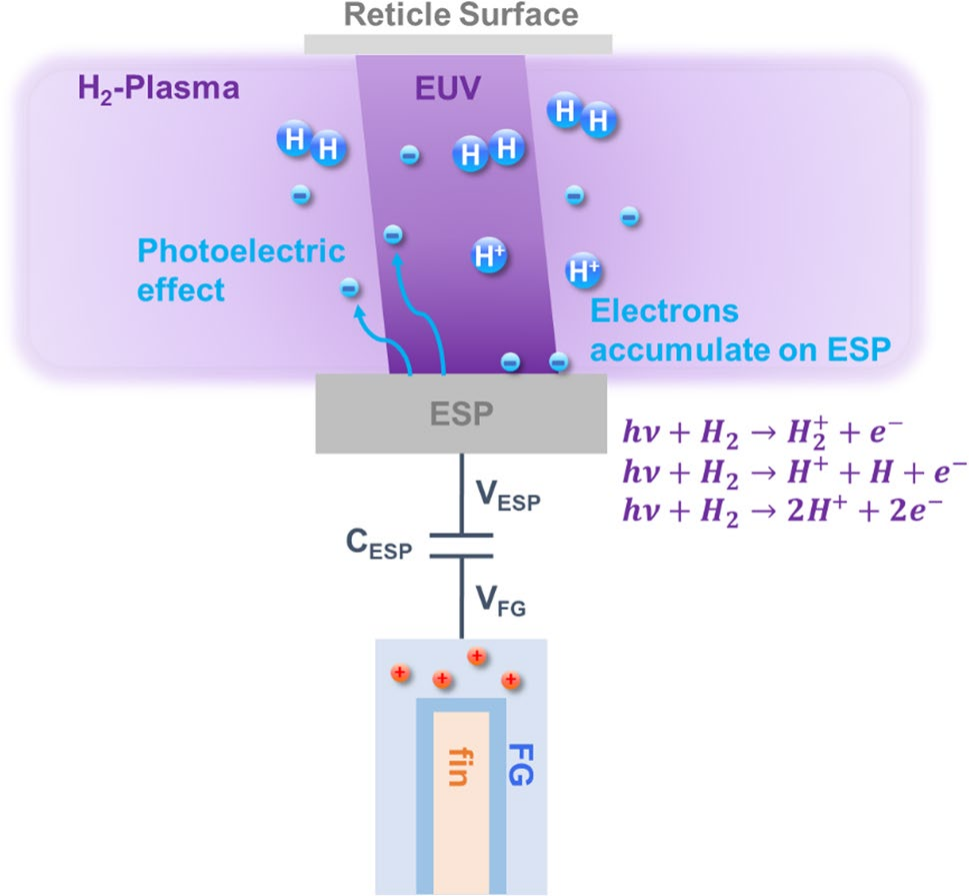
Two different mechanisms—EUV photoelectric effect and H_2_ plasma charging effects—are observed in the EUV system. Charge in FG can be altered as ESP is pulled to either high positive or negative potential charging

Figure 3 illustrates the H_2_ plasma-induced charging effect on the surface metal pad inside a EUV lithography chamber. When the EUV travels through projection optics and reticle, the low-pressure H_2_ background gas in the chamber is excited into H_2_ plasma [24, 25]. When the EUV is projected on ESPs, the electrons are ejected from the surface through the photoelectric effect, leading to a rise in the potential of ESP (V_ESP_) rise when positive charges accumulate on the ESPs. Meanwhile, the negative charge generated by the H_2_ plasma can be collected by the ESP, which causes V_ESP_ to drop accordingly. V_ESP_ is coupled to the potential of FG (V_FG_) by the coupling structure, whose capacitance is C_ESP_. After that, positive charges are drawn into FG through the gate dielectric by Fowler–Nordheim (FN) tunneling [26]. Therefore, the V_th_ of the micro-detector drops, whereas its bit cell current, i.e., BCC, increases under the same read condition. Since the amount of FG charge (Q_FG_) is affected by both EUV exposure and plasma charging effect, EUV exposure experiments were carried out to characterize these two charging events, which co-occur on the ESP during EUV processes.

## Experimental results and discussion

**Fig. 4:**
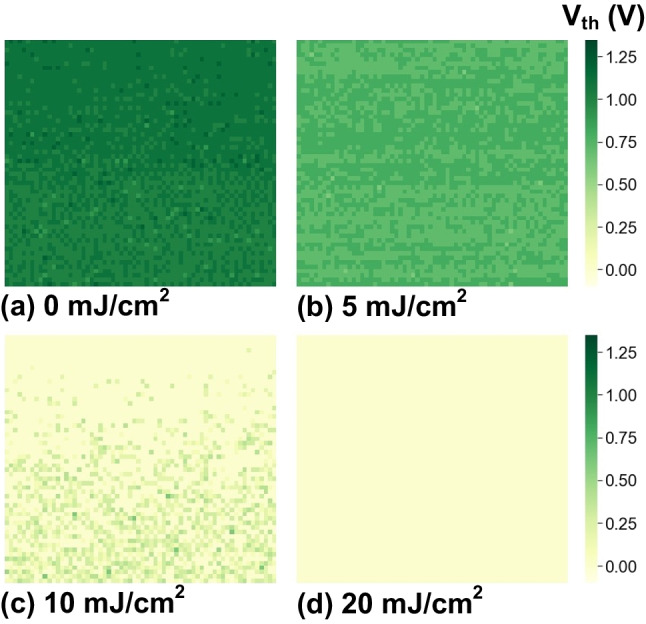
Bitmap of V_th_ of the 4k MDA with array area of 500 μm × 500 μm before (**a**) and after exposure by increasing EUV dosage of **b** 5 mJ/cm^2^, **c** 10 mJ/cm^2^, **d** 20 mJ/cm^2^, respectively

The on-wafer MDAs were sent into the EUV lithography system and were exposed to EUV of different dosages, from 0 to 20 mJ/cm^2^. Figure 4 shows the bitmaps of threshold voltage (V_th_) within the 4k-pixel array with a pixel pitch of 7.7 μm under different EUV exposure intensities. The V_th_ is extracted from the channel current of each pixel from the test detector array using the constant current method. Based on the experimental data, as EUV dosage increases, the measured V_th_ of floating-gate transistors drops as ESPs are charged by the electrons induced by H_2_ plasma, leading to net positive FG charge change. The photoelectric effect increases the voltage of the ESP, leading to an increased attraction to negative charges. As a result, the net charge on ESP becomes negative. When the negative V_ESP_ is coupled to V_FG_, the total charge in the FG increases, causing the V_th_ to decrease.

Before EUV exposure (0 mJ/cm^2^), the bitmap shows a uniform level across the array area of 500 μm × 500 μm, without any failed bit. When the dosage increases to 10 mJ/cm^2^, the V_th_ shift of the upper part is more significant than that of the lower part, which indicates that the local variation in charging of ESP may occur under high hydrogen plasma conditions. The regions with a higher V_th_ shift are more susceptible to PID. The estimated V_ESP_ level projected here can reach more than 8 V under this experiment condition.

**Fig. 5:**
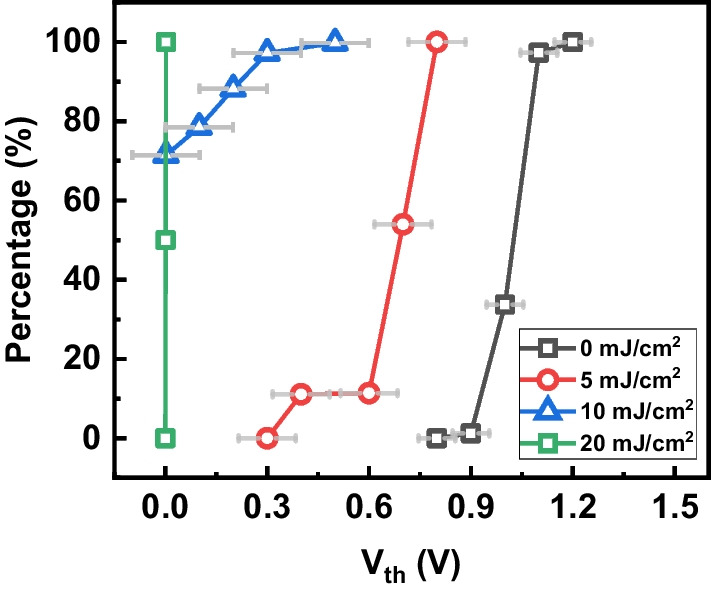
V_th_ distributions within a 4k-pixel MDA. V_th_ drops as the plasma charges ESP after EUV exposure

Furthermore, the V_th_ distributions within the MDA can be analyzed by cumulative percentage, as shown in Fig. 5. Initially, the V_th_ of the MDA is about 1 V, see Fig. 4a, in a tight distribution. As expected, when negative charging of ESPs is dominated by plasma charging in EUV chambers, V_th_ shifts toward negative as the EUV dosage increases. Moreover, the local variations of hydrogen plasma lead to a wider V_th_ spread under high EUV dosage conditions. Finally, the V_th_ drops below zero as Q_FG_ becomes more positive, while the state of Q_FG_ can be reflected by the BCC levels.

Additionally, the exposure time is about 0.1 ms in the experiment, and V_th_ of all cells drop below zero under 20 mJ/cm^2^. In typical scanners, exposure time per field is about 300 ms, and the MDA’s response speed is high enough for *in situ* H_2_-induced plasma detection in lithography systems. Also, the current MDA can detect the hydrogen plasma charging effect resulting from a EUV exposure of 5 mJ/cm^2^ differences, and its sensitivity can be controlled by the designing C_ESP_ of the MDA. When the C_ESP_ increases by using a longer contact slot, the sensitivity can be enhanced accordingly.

While the total response of MDAs combines the plasma charging effect and the photoelectric effect, the V_th_ can be mapped to a EUV exposure dosage. Therefore, the V_th_ shift can reflect the charging effect of H_2_ plasma under different EUV dosages despite the presence of the photoelectric effect.

**Fig. 6:**
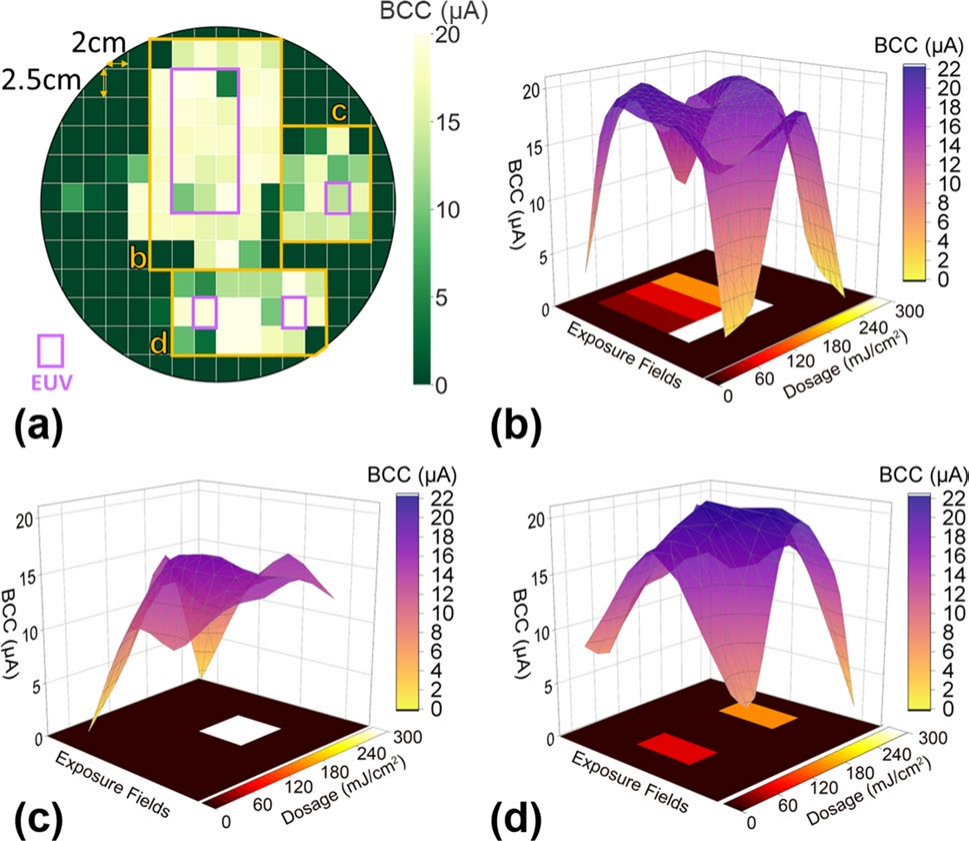
**a** Wafer map of MDA bit cell current (BCC) after EUV exposure. **b**–**d** The bottom plots show the EUV dosage on multiple exposure fields, while the upper plots compare the average BCC from MDA on different parts of the wafer. The BCC saturates under high dosage exposure

Figure 6a shows the average BCC of MDAs on a 12-inch wafer after the EUV exposure, where the dosage ranges from 30 to 300 mJ/cm^2^. Four purple frames on the wafer indicate the reticles exposed to EUV. In Fig. 6b–d, the average BCC level over the whole MDA rises as the V_th_ becomes more negative. Moreover, the MDAs in adjacent exposed reticles are found to be subjected to the electron charging effect due to EUV-induced H_2_ plasma. This suggested that the H_2_ plasma-induced electrons spread wider than the exposed region, leading to more complex charging patterns on wafers, as shown in Fig. 6.

## Conclusions

The EUV-induced H_2_ plasma is analyzed by 4k-pixel on-wafer MDAs in the lithography chamber, and EUV parameters are extracted by the V_th_ and bit cell current from the MDA. The local distribution of plasma can be observed by a single MDA, while the distribution across the wafer can be monitored by MDAs placed in different reticle fields, and the spatial uniformity of plasma can be analyzed. The MDA can become a powerful tool, providing insight observations/on-wafer monitoring of the EUV-induced H_2_ plasma inside future lithography chambers for future process optimization.

## Data Availability

The datasets generated during and/or analyzed during the current study are not publicly available due to the policy of our cooperative research company but are available from the corresponding author on reasonable request.

## Abbreviations

EUV: Extreme ultraviolet
MDA: Micro-detector array
BCC: Bit cell current
ESP: Energy sensing pad
